# Aids to Obstetrics

**Published:** 1888-03

**Authors:** 


*Aids to Obstetrics*. By SAMUEL NALL, B.A., M.B. Cantab., M.R.C.P. Lond.; First Class Nat. Sci. Honours, Cambridge; Schol. in Nat. Sci., St. Bartholomew's Hospital; Late Casualty Physician, Assistant-Demonstrator of Physiology, and Resident Obstetric Assistant, St. Bartholomew's Hospital; etc., etc. Third Edition. London: BALLIÈRE, TINDALL & COX.

As this is an undated book, we have nothing to say about it. We regret this, as the work ought to be a great one, written by an author who, in addition to requiring four lines on the title-page to set forth his qualifications, can also put "etc." twice after his name.

